# Extraneural Soft Tissue Perineurioma of the Oral Cavity: A Rare Case with Medico-Legal Implications and Literature Review

**DOI:** 10.3390/life15091343

**Published:** 2025-08-25

**Authors:** Daniele Pergolini, Mohamed Mohsen, Simona Zaami, Lina De Paola, Federica Rocchetti, Cinzia Angileri, Eduardo Troiani, Cira Rosaria Tiziana Di Gioia, Giulia Coppola, Gaspare Palaia

**Affiliations:** 1 Department of Oral and Maxillofacial Sciences, “Sapienza” University of Rome, Via Caserta 6, 00161 Rome, Italy; daniele.pergolini@uniroma1.it (D.P.); mmohsen3010@gmail.com (M.M.); federica.rocchetti@uniroma1.it (F.R.); angileri.1899997@studenti.uniroma1.it (C.A.); troiani.1831250@studenti.uniroma1.it (E.T.); gaspare.palaia@uniroma1.it (G.P.); 2 Department of Anatomical, Histological, Forensic and Orthopedic Sciences, “Sapienza” University of Rome, 00161 Rome, Italy; lina.depaola@uniroma1.it; 3 Department of Radiological Sciences, Oncology and Pathology, Policlinico Umberto I, “Sapienza” University of Rome, Viale Regina Elena 326, 00161 Rome, Italy; cira.digioia@uniroma1.it (C.R.T.D.G.); giulia.coppola@uniroma1.it (G.C.)

**Keywords:** extraneural perineurioma legal implication, nerve sheath neoplasms, oncology, oral lesions, perineurioma

## Abstract

Perineuriomas are a rare form of peripheral nerve sheath tumors, with occurrences in the oral cavity being exceptionally uncommon. This scarcity underscores the clinical significance of each documented case, as it facilitates enhanced diagnostic precision among oral health professionals. We hereby present a case involving a 68-year-old female patient with an extraneural perineurioma (EPN) located on the mandibular region. A laser-assisted excisional biopsy was performed, and the diagnosis of EPN was confirmed through histopathological examination complemented by immunohistochemical analysis. The lesion was surgically excised, and no recurrence was observed during a one-year follow-up period. Accurate recognition of EPNs in the oral cavity is crucial to prevent unnecessary aggressive surgical interventions. Misdiagnoses may occur in cases of fibromas, neurofibromas, schwannomas, ossifying fibromas, or low-grade malignancies, which can potentially result in overtreatment that may compromise both function and aesthetics. Given the rarity of oral EPN, precise diagnosis and appropriate management are essential to avoid unwarranted invasive procedures and to mitigate potential medico-legal implications originating from misdiagnosis or suboptimal treatment. Ensuring comprehensive informed consent and meticulous documentation is also vital in minimizing medico-legal risks.

## 1. Introduction

Perineurioma (PN) is a rare and typically non-cancerous tumor that arises from perineural cells (PCs) [1,2]. These cells are located between the endoneurium and epineurium and play a role in forming a protective layer around peripheral nerves.

First described by Lazarus and Trombetta in 1978, PN has been slow to gain acceptance. The cause of perineuriomas (PNs) is still debated. Historically, PNs were thought to be non-cancerous growths primarily linked to local trauma or injury [3]. More recently, some authors have suggested that PNs are true benign tumors, linked to changes in chromosome 22. Such alterations can affect a tumor suppressor gene, potentially leading to the clonal proliferation of perineural cells.

There are two subtypes of PNs: intraneural perineurioma (IPN) and EPN. The EPN is the common subtype [4].

EPN, also called soft tissue PN, can arise in a variety of different locations, such as the stomach [5], kidney [6], etc. [7], showing heterogeneous clinical and histopathological features [8,9]. Oral EPNs are uncommon [10]. Cases have been documented in locations such as the mandible, tongue, gums, and lips [11,12].

EPNs are seen in adults of all ages, with a higher incidence in females. They are usually present, as in this instance, as asymptomatic, slow-growing, and well-defined submucosal nodules or masses. Some unusual histological variants of EPN have been described, including reticular, sclerosing, plexiform, and hybrid subtypes [13] Table 1.

IPNs are linked to the enlargement of one or more affected nerves and may lead to sensory and motor impairments [14]. On the other hand, EPNs are usually unrelated to peripheral nerves. They usually appear as a single, asymptomatic nodule or mass, predominantly found in the superficial soft tissues of the limbs and trunk. Both subtypes have an even gender distribution, and they mostly present in middle-aged adults [15].

Intraoral PNs have been rarely mentioned in the literature [5,6]. The aim of this report was to present a case of intraoral EPN, to highlight the clinicopathological spectrum of this unusual tumor.

A PubMed search was conducted for case reports of intraoral NDPs published between 1981 and 2017, and only 18 case reports were available, as shown in Table 1.

**Table 1:** Summary of reported cases of oral extraneural perineurioma (EPN) in the literature (1981–2017) with the following information: demographics, location, size, treatment, and follow-up, based on verified data from PubMed.

No.	Author (year)	Age/Sex	Location	Size (mm)	Treatment	Follow-up/Outcome	Reference
1	Schadel et al., 2019 [1]	20/M	Dorsal Surface of the Tongue	N/R	Surgical Excision	Recurrence and re-excision at 28 months	J Oral Maxillofac Surg. 2019
2	Gomes da Silva et al., 2017 [16]	46/F	Buccal Mucosa	2.5 cm	Surgical Excision	No recurrence, long follow-up	Oral Surg Oral Med Oral Pathol Oral Radiol. 2017
3	Noonan et al., 2010 [17]	17/M	Buccal Mucosa	7–8 mm	Surgical Excision	No recurrence (time not specified)	Head Neck Pathol. 2010
4	Colizza et al., 2010 [18]	17/M	Buccal Mucosa	N/R	Surgical Excision	No recurrence at 8–9 months	Ear Nose Throat J. 2010
5	Kawakami et al., 2012 [19]	43/M	Upper Lip	2.0 × 1.9 cm	Surgical Excision	No recurrence at 16 months	Oral Surg Oral Med Oral Pathol Oral Radiol. 2012
6	Kusama et al., 2003 [20]	31/F	Mandible	N/R	Surgical Excision	N/D	J Nihon Univ Sch Dent. 2003
7	Graadt van Roggen et al., 2001 [13]	42/F	Gingiva	N/R	Surgical Excision	N/D	Am J Surg Pathol. 2001
8	Barrett et al., 2002 [21]	53/M	Mandible	N/R	Surgical Excision	N/D	Oral Oncol. 2002
9	Meer et al., 2003 [22]	46/F	Naso-labial	N/R	Surgical Excision	N/D	Oral Oncol. 2003
10	Damm et al., 2004 [23]	26/F	Tongue	N/R	Surgical Excision	N/D	Oral Surg Oral Med Oral Pathol Oral Radiol Endod. 2004
11	Huguet et al., 2004 [24]	64/M	Mandible	N/R	Surgical Excision	N/D	Oral Oncol Extra. 2004
12	Ide et al., 2004 [25]	59/F	Gingiva	N/R	Surgical Excision	N/D	Oral Oncol. 2004
13	Hornick & Fletcher 2005 [15]	8/F; 44/M	Tongue/Upper lip	N/R	Surgical Excision	N/D	Am J Surg Pathol. 2005
14	Da Cruz Perez et al., 2006 [26]	12/M	Tongue	N/R	Surgical Excision	N/D	Oral Surg Oral Med Oral Pathol Oral Radiol. 2006
15	Dundr et al., 2007 [27]	16/M	Buccal Mucosa	N/R	Surgical Excision	N/D	Pathol Res Pract. 2007
16	Boyanton et al., 2007 [28]	6/F	Tongue	N/R	Surgical Excision	N/D	Arch Pathol Lab Med. 2007
17	Siponen et al., 2007 [29]	19/F	Buccal Mucosa	N/R	Surgical Excision	N/D	Oral Oncol Extra. 2007
18	Tanaka et al., 2007 [30]	34/F	Tongue	N/R	Surgical Excision	N/D	Oral Surg Oral Med Oral Pathol Oral Radiol. 2007

## 2. Case Report

A 68-year-old Caucasian female was referred to the Department of Oral and Maxillofacial Sciences for the evaluation of a slow-growing, painless intraoral swelling.

Her medical history showed that she is a smoker (10 cigarettes/day), has Hashimoto’s thyroiditis, hypertension, type 2 diabetes mellitus, and hypercholesterolemia. She had regular assumption of the following medications: bisoprolol, ramipril, simvastatin, metformin, and levothyroxine.

**Figure 1:**
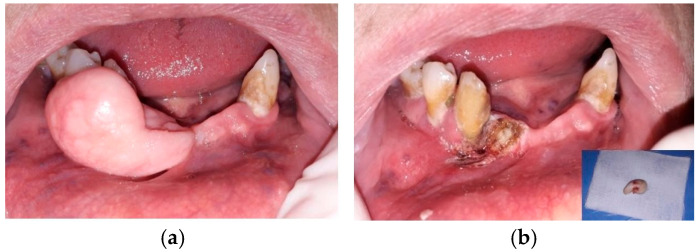
(**a**). Intraoral preoperative aspect of the lesion. (**b**). Intraoral postoperative aspect of the lesion and aspect of the surgical specimen after removal.

Intraoral examination revealed a well-delineated, exophytic, pedunculated, elastic, non-tender mass on the gingival mucosa in the 4.2–4.3 area. The mucosa covering the lesion was similar in color and texture to the surrounding normal mucosa (Figure 1a). The vitality test was positive for tooth 4.3.

Given her multiple chronic health conditions, polypharmacy, and smoking habits, the patient exhibited characteristics of frailty, which is defined as a state of increased vulnerability to stressors due to a decline in physiological reserves.

According to the patient, the lesion had been slowly increasing in size, and there was no history of prior trauma to the area. No cervical lymphadenopathy was clinically evident or palpable.

The differential diagnosis for this lesion includes several benign soft tissue tumors and reactive lesions, such as neurofibromas, schwannomas, pyogenic granulomas, traumatic neuroma, myofibroma, and benign fibrous histiocytoma.

Blood chemistry and coagulation tests were prescribed prior to scheduling an excisional biopsy. The biopsy was performed using a blue light diode laser (Eltech K-Laser srl, Treviso, Italy), with a wavelength of 445 ± 5 nm, set at 2.5 Watts in Continuous Wave (CW) mode, utilizing a 320 μm surgical optical fiber and a fluence of 3100 J/cm^2^, according to the device settings (Figure 1b).

Local anesthesia was administered with 1.8 mL of mepivacaine solution (MEPIVACAINA PIERREL^®^, 30 mg/mL, injection solution 1.8 mL, Pierrel Spa, Milan, Italy) prior to the beginning of the surgical intervention. The sample was sent to the pathologist for histological evaluation and diagnosis.

## 3. Results

Gross examination revealed that the surgically excised mass was circumscribed but not encapsulated, measuring 2.5 cm × 2 cm × 0.5 cm.

Microscopic examination revealed a circumscribed but unencapsulated proliferation of spindle cells with eosinophilic cytoplasm and bland, elongated nuclei. The stroma turned out to be fibromyxoid in some areas.

Cell pleomorphism, mitosis, and necrosis were absent. Scattered vascular elements were observed throughout, with whorls of spindle cells arranged in a vague perivascular configuration.

**Figure 2:**
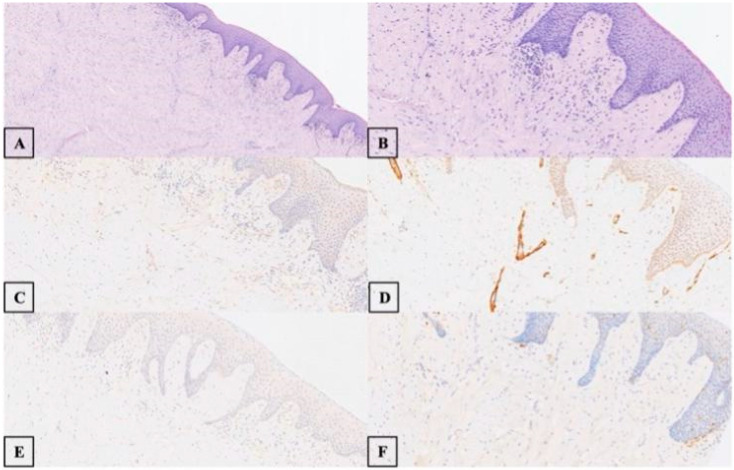
Histologic examination revealed a circumscribed but unencapsulated submucosal hypocellular nodule composed of small spindle to ovoid cells with indistinct cell borders and long, streamer-like cell processes arranged in a short fascicular pattern within a dense sclerotic and focally fibromyxoid matrix ((**A**) [4× magnification] and (**B**) [10× magnification]). Cell pleomorphism, mitosis, and necrosis were absent. The overlying epithelium appeared unremarkable. Immunohistochemical analysis showed strong, focal cytoplasmic immunoreactivity in the neoplastic cells for GLUT-1 ((**C**) [10× magnification]) and weak, focal cytoplasmic immunoreactivity for AML ((**D**) [10× magnification]). The neoplastic cells were negative for Desmin ((**E**) [10× magnification]) and S100 ((**F**) [10× magnification]).

Immunohistochemistry demonstrated strong, focal reactivity for glucose transporter 1 (GLUT-1) and weak reactivity for epithelial membrane antigen (EMA). The tumor cells were negative for S-100 protein, desmin, and muscle-specific actin (MSA) (Figure 2). We used only GLUT-1 and S100 as the main markers for the diagnosis of perineurioma because these markers are generally considered reliable for this diagnosis. Furthermore, Claudin-1 is typically employed only in selected cases or for further studies when there are uncertainties, due to limited laboratory resources.

**Figure 3:**
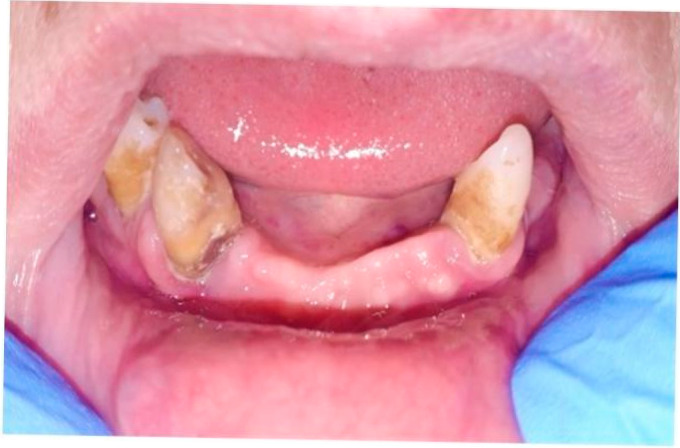
One-year follow-up.

In contrast, schwannomas are usually well-encapsulated tumors exhibiting alternating hypercellular (Antoni A) and hypocellular (Antoni B) areas. Antoni A areas show densely packed spindle cells with nuclear palisading (Verocay bodies), which are absent in the described lesion. Schwannomas typically lack a fibromyxoid stroma and have more uniform architecture [31]. A final diagnosis of EPN was established. The patient had an uneventful postsurgical recovery and showed no signs of recurrence at a 1-year follow-up appointment (Figure 3).

## 4. Discussion

PN is a benign neoplasm; however, some rare cases of malignant PNs have been reported and described as a cancerous tumor that originates in the peripheral nerve sheath and exhibits characteristics like perineurial cells [13]. Recently, in English-language literature, a case of malignant PN has been described in a 24-year-old Japanese male on the lateral border of the tongue. The patient was subjected to partial glossectomy without recurrence after two years of follow-up [14,15].

The histological diagnosis of malignant PN is achieved by observing the presence of an infiltrative growth pattern, significant cytologic atypia, the presence of necrosis, and high mitotic activity [16,17].

Oral PN is a rare entity that may pose a diagnostic challenge to clinicians. A full awareness of its characteristic histopathologic features and immunohistochemical profile is essential in order to distinguish this unique neoplasm from other spindle cell lesions that may be considered in the differential diagnosis [18,19].

Perineurial cells make up a small portion of many abnormal or tumor-like growths in the peripheral nerve sheath, such as neurilemmomas, neurofibromas, traumatic neuromas, and solitary circumscribed neuromas [13,14]. In contrast, they are the main or sole component in the rare benign tumor of the peripheral nerve sheath called perineurioma [15].

PNs are benign neoplasms linked to alterations in chromosome 22 that affect tumor suppressor genes, leading to the clonal growth of perineural cells [20].

EPN, a type of PN, is rare in the oral cavity but can occur in areas like the mandible, tongue, gingiva, and lips [21]. Due to the rarity of PN in the oral cavity, oral clinicians and oral surgeons may not be well-acquainted with this type of tumor [22,23].

### 4.1. Differential Diagnosis

Oral EPNs often appear as asymptomatic, slow-growing, and well-defined soft tissue lesions with a higher prevalence in females. Clinically, they may be misdiagnosed as neurofibromas, schwannomas, or pyogenic granulomas [24,25].

Initial diagnostic hypotheses can include traumatic neuroma, myofibroma, and benign fibrous histiocytoma. Accurate differential diagnosis is critical to avoid unnecessary aggressive treatments.

### 4.2. Histology and Immunohistochemistry

The diagnosis of EPN requires the correlation of the histomorphologic findings with immunohistochemical or ultrastructural evidence of perineurial differentiation [15].

In the present case, histological examination pointed to a characteristic pattern in which a hypocellular submucosal nodule was composed of small spindle to ovoid cells with indistinct borders and long cytoplasmic processes, arranged in fascicles within a dense, partially fibromyxoid matrix, with no evidence of mitotic activity or necrosis. Immunohistochemical analysis further demonstrated diffuse cytoplasmic positivity for GLUT-1 and focal positivity for AML. Ultrastructural or immunohistochemical confirmation of perineurial differentiation is fundamental for the diagnosis of soft tissue perineurioma [15,25,26].

The histological and immunohistochemical findings, characterized by a typical morphological pattern and positivity for GLUT-1, support the diagnosis of soft tissue perineurioma. The negativity for S100 and Desmin excludes other neurogenic or muscular differentiation neoplasms, while the absence of mitosis and necrosis confirms the benign nature of the tumor. These findings, integrated with ultrastructural analysis, highlight a perineurial differentiation process.

The most common morphological feature of soft tissue perineuriomas is the presence of uniform, bland spindle or epithelioid neoplastic cells. These cells are typically arranged in loose fascicular, storiform, or whorled patterns. The nuclei are ovoid to elongated in shape, with subtle nucleoli. The stroma may appear significantly myxoid, collagenous, or hyalinized, though mitotic figures are uncommon [11].

Although not entirely definitive, the immunoreactivity of the neoplastic cells for perineural cell markers such as EMA [27,28,29], Claudin-1 [30], and human erythrocyte glucose transporter-1 (GLUT-1) can help diagnose PN [31].

### 4.3. Treatment

The treatment of perineurioma is primarily surgical. Complete local surgical resection with clear margins is the main therapeutic approach to remove the tumor and is considered curative, as perineuriomas are generally benign [2]. Overly aggressive treatments are not advisable unless complications arise. Despite the risk of recurrence, the long-term prognosis is generally favorable. Postoperative monitoring is important, though radiotherapy may be considered in some instances, if the resection is incomplete.

In our case, as in many others [1,15,16,17,18,19,20,21,22,23,24,25,26,27,28,29,30], the use of a 445 nm laser helped in the complete hemostasis and offers several benefits [29,32], such as cleaning the surgical area, reducing postoperative bleeding [27,28], and greatly minimizing inflammation and pain after the procedure [15,24,25,26,27,28,29] In the past, some studies reported the possible risk of alteration in histological analysis due to the thermal effect after oral laser biopsy. Recently, studies confirmed the absence of this risk in the case of the correct use of laser by a skilled operator [30,31].

### 4.4. Medico Legal Framework

Despite the rarity of the case, the medico-legal implications are certainly not negligible [33]. They mainly concern clinical management, the correct diagnosis, and the treatment of the patient. Oral EPN cases, due to their rarity, can lead to misdiagnosis or delayed diagnosis, which may result in inadequate treatments, such as overly aggressive or, conversely, incomplete surgical excisions [32].

If the healthcare provider fails to properly recognize EPN, this could bring about a higher risk of damage to surrounding structures, such as the inferior alveolar nerve [34], or tumor recurrence, necessitating more extensive surgical excisions, aesthetic and functional disfigurement of the masticatory apparatus, or tumor progression [35,36].

Any permanent damage to the patient could carry potential legal implications for the physician, including issues related to incorrect or inadequate oncological counseling [20,37,38,39,40]. Physicians are bound to provide their patients with thorough information as to all available therapeutic options, also by elaborating on the risks, benefits, and potential complications [41,42,43,44,45]. Informed consent, like many other therapeutic options, needs to be clear, conscious, voluntary, timely, and unambiguous [46,47,48,49]. Without such core features, the physician could be accused of violating the patient’s right to self-determination [50,51,52,53].

It is essential that the clinical record and all the documentation related to the case and the patient are clear and accurate. It is crucial that all stages of the diagnostic and therapeutic process are well documented [54]. This should also include the results of histological and immunohistochemical examinations, communication with the patient regarding the diagnosis and proposed therapeutic options—counseling—as well as, naturally, the documentation of the postoperative follow-up and the medical instructions given to the patient for the care and hygiene of the surgical site, to avoid unjust accusations.

Furthermore, failure to adhere to treatment and follow-up guidelines could in itself entail a medico-legal risk [55,56]. In fact, the law stipulates that the doctor’s liability cannot be proven and linked to any potential harm if the procedures identified as guidelines or best clinical practices for that given condition have been adhered to.

In any case, accuracy in diagnosis, proper and complete information for the patient, and adherence to therapeutic guidelines are mandatory to avoid legal action and to treat the patient in the best possible way, in line with the principle “primum non nocere” [32,57,58].

In conclusion, from our research, it can be inferred that medico-legal implications revolve around the correct diagnostic and therapeutic management of the case, particularly in rare cases, the importance of informing and involving the patient in the decision-making process and remembering that the time spent on informing the patient is also, by law, considered part of the care process. In cases of rare diseases, managing follow-up management and implementation are particularly meaningful and relevant.

Ultimately, accurate management of the entire diagnostic and therapeutic process can significantly lower the risk of legal issues for healthcare providers [35,36,37] and physical and psychological harm to the patient [45,54,55]. Additionally, with the advent of new diagnostic technologies, such as artificial intelligence (AI) [56,57], the accuracy and efficiency of dental and medical diagnoses, especially in rare cases like the one described by the authors, could be significantly improved. AI could help identify patterns once provided with large amounts of data, as already happens in many other branches of medicine [57], toxicology [58,59,60,61], and in both clinical and forensic dentistry [62,63], which might be difficult for human professionals to recognize [64]. AI systems could provide more precise and faster diagnoses, which could result in better outcomes for patients and reduce the risk of misdiagnoses [65], in turn leading to fewer disputes for healthcare institutions and providers alike.

The importance of the medico-legal aspect might initially seem marginal or not significantly different from that of many other oncological cases. This case was noteworthy not only because of the medico-legal implications common to any cancer case, but also, perhaps even more so, because of the rarity of the condition it presented.

Nowadays, litigation against healthcare professionals, particularly in countries that do not adopt a no-fault compensation system [38,39,40,41,42,66], has become a considerable issue. This makes it even more essential, especially in rare pathological cases, to emphasize the bureaucratic and documentation aspects, which might otherwise be overlooked or treated as secondary.

Therefore, in rare oncological pathologies, the medico-legal implications are of even greater relevance. Relatively limited clinical experience and the absence of standardized diagnostic and therapeutic protocols can increase the risk of misdiagnosis, delayed treatment, or suboptimal care. In such contexts, thorough medical documentation, transparent communication with the patient, and strict adherence to informed consent procedures become not only good clinical practice but also critical safeguards against legal disputes [35,57]. Addressing these aspects proactively can help protect both the patient’s rights and uphold the healthcare provider’s legal tenability, shielding them from possible negligence-based malpractice allegations and lawsuits.

### 4.5. Limitations

This work is subject to certain limitations, including the nature of a case report, which does not allow for generalization of the results. The patient was reviewed at one year, which represents a relatively short follow-up period; she has, however, been enrolled in a follow-up program and will be reviewed in the coming years to monitor for possible late recurrences. Additionally, the one-year follow-up does not entirely rule out the possibility of late recurrences, which we plan to investigate in the future. The rarity of EPN in the oral region limits the opportunity for comparison with other cases in the literature; however, this also represents one of the strengths of our article. Another limitation is the absence of radiological images, which we fully acknowledge would have been of great value; nonetheless, we considered it important to report this case so that it may be clear that such imaging should be routinely obtained to better characterize rare presentations like the one we describe.

## 5. Conclusions

In conclusion, although extraneural perineuriomas are rare, their occurrence in the oral cavity underscores the importance of recognizing this entity in the differential diagnosis of oral lesions.

The clinical and histopathological features of EPN are often subtle, but with proper diagnostic techniques, such as histology and immunohistochemistry, a correct diagnosis can be achieved.

An accurate diagnosis is also instrumental in minimizing medico-legal risks and professional liability claims for unnecessary or inappropriate treatments. The favorable prognosis associated with complete surgical resection further highlights the importance of early detection and appropriate management in ensuring positive patient outcomes.

## Data Availability

All data generated or analyzed during this study are included in this published article and are also available from the corresponding author upon reasonable request.
